# Cannabis and psychosis

**DOI:** 10.1192/j.eurpsy.2026.10410

**Published:** 2026-07-31

**Authors:** J. G. Bramness

**Affiliations:** Oslo University Hospital, Oslo, Norway

## Abstract

**Abstract:**

This presentation reviews current evidence on the relationship between cannabis use and psychosis with special emphasis on cannabis induced psychosis, schizophrenia and the transition between the two. Cannabis has become increasingly potent over time, with THC concentrations rising dramatically, and this change is accompanied by a growing body of evidence linking cannabis to a range of psychiatric outcomes. Although cannabis is generally considered less harmful than alcohol, it nonetheless contributes to significant mental health risks. Epidemiological data consistently show associations between cannabis use and depression, anxiety, cognitive impairment, and, the primary focus of this lecture, psychotic disorders. Both heavy use and early onset substantially increase these risks, especially among vulnerable individuals.

Acute cannabis-induced psychosis is well documented and typically presents with confusion, hallucinations, emotional instability, depersonalization, and paranoid ideation. The condition can resemble acute schizophrenic psychosis, and symptom patterns are quite similar. While most cases are resolved within days, an acute psychotic episode represents an important clinical warning sign. Some debate persists about whether cannabis can cause persistent psychosis, but accumulating findings indicate that cannabis so consistently triggers schizophrenia in vulnerable individuals, that we mostly look at it as a separate causal factor. Large cohort and genetic studies suggest that the association reflects both shared vulnerability and a direct causal contribution of cannabis. Dose–response relationships have been observed for frequency of use, potency, and age at initiation.

Several candidate genes—including COMT, DRD2, and FAAH—appear to interact with cannabis to increase psychosis risk, but findings remain inconsistent. Individuals with schizophrenia who use cannabis experience poorer clinical outcomes, including reduced medication adherence, more frequent relapses, and increased hospitalizations. Studies show rising treatment-seeking for cannabis-induced psychosis paralleling increased potency of cannabis products.

The transition from cannabis-induced psychosis to schizophrenia is a central clinical concern. Recent meta-analytic evidence indicates that about 20% of people with cannabis-induced psychosis later develop a schizophrenia-spectrum disorder, underscoring the long-term implications. Diagnostic and legal frameworks complicate assessment, as time-based criteria in DSM and ICD attempt to separate substance-induced from primary psychosis, despite substantial clinical overlap. These distinctions have direct consequences for treatment, follow-up, and legal responsibility.

Overall, the evidence strongly supports cannabis as a risk factor for psychosis—particularly for early, frequent, and high-potency use—and highlights the importance of identifying vulnerable individuals and providing early, appropriate intervention.

**Disclosure of Interest:**

None Declared

